# The IL-10 and IFN-γ pathways are essential to the potent immunosuppressive activity of cultured CD8^+ ^NKT-like cells

**DOI:** 10.1186/gb-2008-9-7-r119

**Published:** 2008-07-29

**Authors:** Li Zhou, Hongjie Wang, Xing Zhong, Yulan Jin, Qing-Sheng Mi, Ashok Sharma, Richard A McIndoe, Nikhil Garge, Robert Podolsky, Jin-Xiong She

**Affiliations:** 1 Center for Biotechnology and Genomic Medicine, Medical College of Georgia, 15th Street, Augusta, GA 30912, USA; 2 Department of Pathology, Medical College of Georgia, 15th Street, Augusta, GA 30912, USA; 3 Department of Medicine, Medical College of Georgia, 15th Street, Augusta, GA 30912, USA

## Abstract

**Background:**

CD8^+ ^NKT-like cells are naturally occurring but rare T cells that express both T cell and natural killer cell markers. These cells may play key roles in establishing tolerance to self-antigens; however, their mechanism of action and molecular profiles are poorly characterized due to their low frequencies. We developed an efficient *in vitro *protocol to produce CD8^+ ^T cells that express natural killer cell markers (CD8^+ ^NKT-like cells) and extensively characterized their functional and molecular phenotypes using a variety of techniques.

**Results:**

Large numbers of CD8^+ ^NKT-like cells were obtained through culture of naïve CD8^+ ^T cells using anti-CD3/anti-CD28-coated beads and high dose IL-2. These cells possess potent activity in suppressing the proliferation of naïve responder T cells. Gene expression profiling suggests that the cultured CD8^+ ^NKT-like cells and the naïve CD8^+ ^T cells differ by more than 2-fold for about 3,000 genes, among which 314 are upregulated by more than 5-fold and 113 are upregulated by more than 10-fold in the CD8^+ ^NKT-like cells. A large proportion of the highly upregulated genes are soluble factors or surface markers that have previously been implicated in immune suppression or are likely to possess immunosuppressive properties. Many of these genes are regulated by two key cytokines, IL-10 and IFN-γ. The immunosuppressive activities of cells cultured from IL-10^-/- ^and IFN-γ^-/- ^mice are reduced by about 70% and about 50%, respectively, compared to wild-type mice.

**Conclusion:**

Immunosuppressive CD8^+ ^NKT-like cells can be efficiently produced and their immunosuppressive activity is related to many surface and soluble molecules regulated by IL-10 and IFN-γ.

## Abstract 2

Global gene expression profiling of *in vitro* cultured CD8^+ ^T cells that express natural killer cell markers revealed differential expression of about 3,000 genes between these cells and naïve CD8^+ ^T cells.

## Background

T cells comprise a heterogeneous population of cells that have different phenotypes and functions. The primary function of T cells is to mount an immune response against invading pathogens, but some T cells can mount an immune response against self-proteins and thus cause a variety of autoimmune diseases if they are not properly controlled by a T cell population known as regulatory T cells (Treg cells). There are several well defined Treg cell subsets and the best studied is the CD4^+^CD25^+ ^Treg cells, which possess potent activity in suppressing the proliferation of both CD4^+ ^and CD8^+ ^effector T cells *in vitro *and *in vivo*. Certain CD8^+ ^T cells have also been recognized to have suppressive function but the CD8^+ ^Treg is poorly defined. T cells with natural killer (NK) cell activity have been identified in both mice and humans [1-4] and these cells are referred to as NKT cells. Murine NKT cells express phenotypic markers that are typically found on T cells, such as CD3 and the αβ T-cell receptor (TCR), and markers for NK cells, such as NK1.1 and DX5 [5]. Two major NKT cell populations have been recognized in mice [6,7]. The first population is the well-characterized invariant NKT (*i*NKT) cells that express invariant Vα14-Jα18 TCR in mice [8-10]. These *i*NKT cells are restricted by the major histocompatibility complex (MHC) class I-like molecule Cd1d and recognize glycolipid antigen α-galactosylceramide, a synthetic variant of a murine sponge-derived glycolipid [8,11]. These *i*NKT cells produce large amounts of interleukin (IL)-4 and interferon (IFN)-γ upon activation and have been shown to play a critical role in regulating the immune response [8,11]. The second population of NKT cells expresses a variable TCR repertoire and is not restricted by Cd1d. These NKT cells express mainly CD8 or are negative for both CD8 and CD4 [6]. The whole αβTCR^+^NK1.1^+ ^NKT population represents 1-2% of splenocytes in B6 mice, and, of these cells, approximately 20% are CD8^+ ^[6]. It has been shown that neonatal tolerance is associated with increased CD8^+ ^NKT-like cells, suggesting that CD8^+ ^NKT-like cells may have immunoregulatory properties [12].

Due to the very low frequency of the CD8^+ ^NKT-like cells, their function and the molecular mechanism underlying their function are poorly understood. Therefore, a number of investigators have attempted to develop *in vitro *and *in vivo *expansion protocols to investigate these rare cells. The Cd1d-independent CD8^+ ^NKT-like cells are increased in certain genetically manipulated mice. For example, three different MHC class I-restricted TCR-transgenic mouse strains (OT-I, P14 and H-Y) contain higher but still low frequencies of transgenic CD8^+ ^T cells that co-express NK cell marker NK1.1 [13]. These transgenic CD8^+ ^NKT-like cells are endowed with effector properties, such as cytokine production and antigen-specific cytotoxicity. Tumor-bearing C57BL/6 mice were shown to have a population of NKT cells that co-express CD8 and NK1.1 [14]. These cells can be maintained in long-term culture with IL-4 but produce large amounts of IFN-γ following activation. These CD8^+ ^NKT-like cells show a potent NK-like cytotoxic activity against multiple tumor targets and their cytotoxic activity is Cd1d-independent [14]. CD8^+ ^cells with NK phenotype can also be expanded *in vitro *using a culture condition that includes IFN-γ, anti-CD3 and IL-2 [15]. Such expanded CD8^+ ^NKT-like cells can efficiently kill tumor cells *in vitro *and *in vivo *but have limited capacity to cause graft-versus-host disease [15]. However, the amplification efficiency for these cells is variable and slight changes in culture conditions may result in cells with very different phenotypes and functions. Cell culture with anti-CD3/anti-CD28-coated beads and high dose IL-2 was previously shown to expand CD4^+ ^Treg cells that can suppress the proliferation of responder T cells and prevent the development of autoimmune diseases in certain models [16,17]. Using a similar protocol, we can efficiently produce, from the total splenic CD8^+ ^T cell population, large numbers of CD8^+ ^T cells that co-express various NK markers. These cells are therefore referred to as CD8^+ ^NKT-like cells. We demonstrate that these cells possess potent immunosuppressive activity and report the molecular profiles of these cells assayed using microarray analysis coupled with multiple confirmation techniques, including RT-PCR, enzyme-linked immunosorbent assay (ELISA) and flow cytometry. Guided by the genomic information, we further demonstrate that IL-10 and IFN-γ are two key pathways implicated in the function of these immunosuppressive CD8^+ ^NKT-like cells.

## Results

### *In vitro *culture of CD8^+ ^T cells

**Figure 1:**
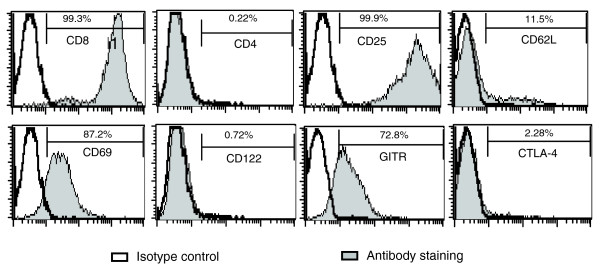
Surface marker expression of cultured CD8^+ ^T cells. The expression profiles of CD8, CD4, CD25, CD62L, CD69, CD122, GITR and CTLA-4 were analyzed by flow cytometory in the tenth day of culture for CD8^+ ^T cells.

*In vitro *cultures with anti-CD3/anti-CD28-coated beads in the presence of high dose IL-2 can efficiently expand CD4^+^CD25^+ ^Treg cells that suppress the proliferation of effector T cells. However, the small number of natural CD4^+ ^Treg cells available for expansion limits the use of this approach. Therefore, we attempted to obtain Treg cells from the more abundant total CD4^+ ^and CD8^+ ^T cell populations from the mouse spleen. Freshly purified splenic CD8^+ ^or Mo-Flow sorted CD4^+ ^T cells from 7-8-week old mice were cultured with an expansion protocol consisting of anti-CD3/anti-CD28-coated beads and high dose IL-2. By the end of the 10-13 days of expansion, the number of cells had generally increased by over 1,000-fold. The cultured cells were phenotyped for a number of surface markers (Figure 1). The vast majority of the cultured cells from CD8^+ ^T cells were positive for CD8 (>95%) and the activation marker CD25 (98%) at the end of the culture. Consistent with the activation of these cells, the percentages of CD62L^+ ^cells gradually decreased and became very low near the end of the culture (around 10%). Similarly, the culture conditions can efficiently expand CD4^+ ^T cells. At the end of the culture, the cultured cells remained CD4^+ ^(97%) and became positive for the activation marker CD25 (99%).

### Cultured CD8^+ ^T cells possess potent immunosuppressive properties

**Figure 2:**
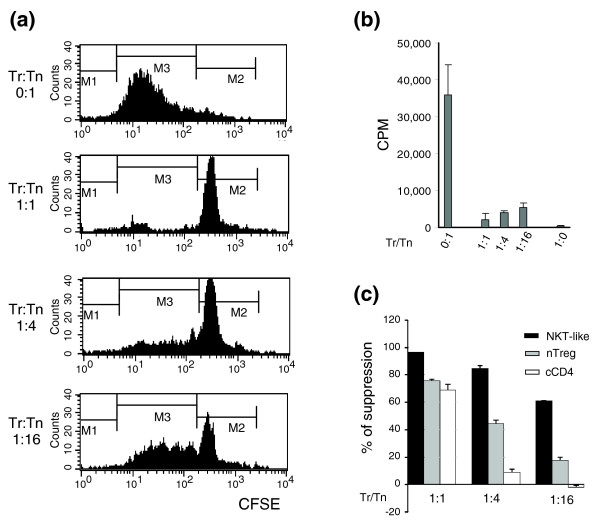
Cultured CD8^+ ^T cells suppress naïve T cell proliferation. **(a) **Dose-dependent suppression of CD4^+^CD25^- ^responder T cells by cultured CD8^+ ^T cells. CFSE-labeled CD4^+^CD25^- ^naïve T cells (Tn) isolated from B6 spleens were stimulated with anti-CD3 (1.5 μg/ml) in the presence of irradiated splenic APCs with graded numbers of cultured CD8^+ ^T cells (Tr). After 72 h in the culture, CFSE dilution in the responder CD4^+ ^T cells was analyzed by flow cytometry. T cells in the M2 zone are undivided cells and T cells in the M3 zone with lower CFSE are divided cells. Data are representative of five independent experiments. **(b) **Naïve CD4^+^CD25^- ^splenic T cells were cultured in the same condition as shown in (a). The cultures were pulsed with 1 μCi/well [^3^H]thymidine at 72 h and the level of proliferation was assessed by [^3^H]thymidine incorporation in the last 16 h of culture. **(c) **Cultured CD8^+ ^T cells (NKT-like), freshly isolated CD4^+^CD25^+ ^Treg cells and cultured CD4^+ ^cells (cCD4) were compared for their ability to suppress the proliferation of CD4^+^CD25^- ^responder T cells. Data are presented as percentage of suppression based on the CFSE dilution with standard deviation. ANOVA test suggests that the suppressive ability is significantly different between these cells (*p *< 10^-6^).

The cultured CD8^+ ^and CD4^+ ^T cells were tested for their ability to inhibit the proliferation of CD4^+^CD25^- ^naïve T cells (Tn cells) using two different *in vitro *suppression assays. In the first assay, the naïve T cells were labeled with carboxyfluorescein succinimidyl ester (CFSE) and T cell proliferation was assessed by the dilution of CFSE signal using fluorescence-activated cell sorting (FACS) analysis. As shown in Figure 2a, the cultured CD8^+ ^T cells efficiently suppressed proliferation of naïve CD4^+^CD25^- ^T cells. The suppressive activity of the cultured CD8^+ ^T cells is dose-dependent and strong suppression can be seen at the 1:16 expanded CD8^+ ^T to Tn cell ratio (Tr/Tn; Figure 2b). In the second suppression assay, T cell proliferation was measured by incorporation of [^3^H]thymidine. As shown in Figure 2c, the dose-dependent suppression activity of the CD8^+ ^T cells was confirmed. Furthermore, the cultured CD8^+ ^T cells did not proliferate in response to anti-CD3 and antigen presenting cell (APC) stimulation. This anergic phenotype is consistent with the observation on CD4^+^CD25^+ ^Treg cells [18,19]. Finally, the cultured CD8^+ ^cells appeared to suppress better than freshly isolated CD4^+^CD25^+ ^Treg cells (Figure 2c; *p *< 10^-6^). The cultured CD4^+ ^T cells also had some suppressive function at the high Tr/Tn ratio of 1:1, while the suppressive activity for the cells gradually became undetectable, suggesting that the suppressive activity of the cultured CD8^+ ^T cells was much higher than the CD4^+ ^T cells cultured under the same conditions (Figure 1c). Therefore, most subsequent studies focused on the phenotype of the cultured CD8^+ ^T cells.

### Gene expression profiles of cultured CD8^+ ^T cells

**Figure 3:**
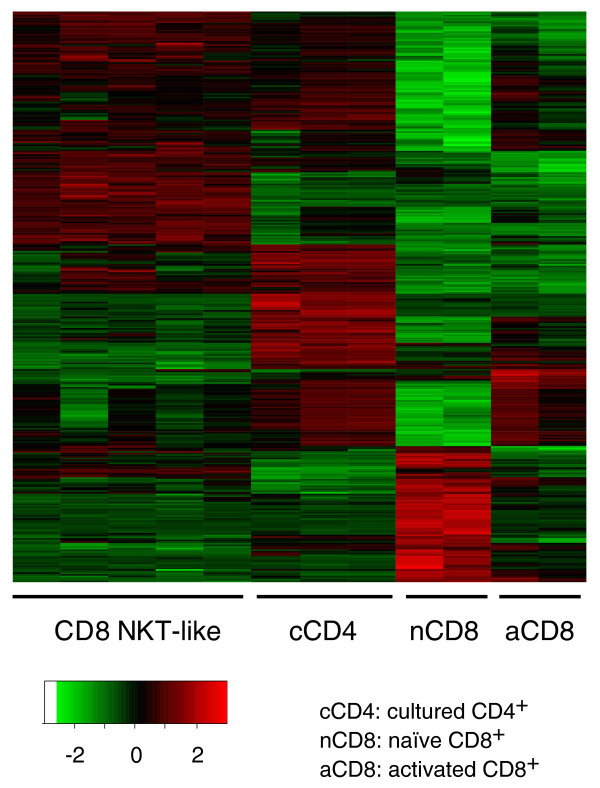
Heat map for genes differentially expressed among the four groups of T cells. Only those genes with a FDR (q) ≤0.01 and fold change ≥5 are included in this map. Data for each gene are standardized separately before being plotted, as is standard in drawing heat maps, so that all genes have a similar scale and the relative differences for all genes can be visualized on a single plot.

To gain further insight into the phenotypes and functions of the cultured CD8^+ ^and CD4^+ ^T cells, we carried out microarray analyses using Affymetrix GeneChips that cover the whole mouse transcriptome (>45,000 transcripts). Five independent cultures of CD8^+ ^T cells and three independent cultures of CD4^+ ^T cells as well as two groups of control cells were included in the microarray analysis. The first group of control cells included two freshly isolated naïve CD8^+ ^T cells and the second control group consisted of two CD8^+ ^T cells activated by a low dose of soluble anti-CD3 and anti-CD28 (activation protocol). Naïve CD8^+ ^T cells as well as activated CD8^+ ^T cells do not possess suppression function. This data set was analyzed as described in Materials and methods and the results are summarized in Table 1. As expected, the expression of thousands of genes was changed by the expansion protocol and the activation protocol compared to naïve CD8^+ ^T cells (Figure 3). Surprisingly, over 100 genes were changed by >10-fold and a few dozen genes were changed by 40- to 800-fold in the cultured CD8^+ ^and CD4^+ ^T cells compared to naïve CD8^+ ^T cells.

**Table 1:** Summary of differentially expressed genes*

Fold change	NKT/nCD8	cCD4/nCD8	aCD8/nCD8	NKT/cCD4	NKT/aCD8	cCD4/aCD8
>10 fold up	113	126	40	26	64	36
5-10 fold up	201	300	27	46	67	47
2-5 fold up	1,742	1,647	3	161	190	47
2-5 fold down	681	693	6	243	76	4
5-10 fold down	100	100	35	59	24	15
>10 fold down	56	59	36	32	15	9

*Only those genes with q-values <0.01 are included in this table. NKT, CD8^+ ^NKT-like cells; nCD8, naïve CD8^+ ^T cells; cCD4, cultured CD4^+ ^T cells; aCD8, activated CD8^+ ^T cells.

To elucidate the molecular basis of the function of the cultured CD8^+ ^T cells, we functionally annotated the 314 genes with >5-fold differences (including 113 genes with >10-fold differences) between the cultured and naïve CD8^+ ^T cells (Table 2). The largest group of differentially expressed genes (17% for >5-fold difference and 31% for >10-fold difference) is, as expected, involved in immunity and defense. The genes with >10-fold differences are enriched by 6-fold compared to the frequency of this functional group in the genome (*p *= 7.7 × 10^-15^). Other significantly enriched gene groups with considerable interest include those involved in apoptosis, cell cycle, cell proliferation and differentiation, and cell adhesion (Table 2). Twenty-three cell cycle genes were upregulated by >5-fold, including 11 genes that were upregulated by >10-fold in the cultured CD8^+ ^T cells (Table 2). Twenty-one genes in the cell proliferation and differentiation category were upregulated and twenty-five upregulated genes belong to the apoptosis group. A number of these genes were selected for confirmation using a combination of real-time RT-PCR, flow cytometry and ELISA. All selected genes have been confirmed and will be discussed in more detail later.

**Table 2:** Major biological processes modified in the cultured CD8^+ ^NKT-like cells

	≥5 fold (314)				≥10 fold (113)			
		
Biological process	Number of genes	% genes	*p*-value	OR	Number of genes	% genes	*p*-value	OR
Immunity and defense	56	17.80%	2.0E-11	3.1	35	31.00%	7.7E-15	6.3
Apoptosis	25	8.00%	8.8E-10	4.8	19	16.80%	2.2E-13	11.3
Lipid, fatty acid metabolism	25	8.00%	7.5E-06	2.9	6	5.30%	0.1161	1.9
Signal transduction	74	23.60%	3.4E-05	1.8	28	24.80%	0.0041	1.9
Cell structure and motility	25	8.00%	0.0004	2.2	11	9.70%	0.0036	2.8
Cell cycle	23	7.30%	0.0004	2.3	11	9.70%	0.0015	3.1
Oncogenesis	14	4.50%	0.0005	3.0	8	7.10%	0.0004	4.9
Protein metabolism and modification	60	19.10%	0.0009	1.6	23	20.40%	0.0152	1.8
Cell proliferation and differentiation	21	6.70%	0.0023	2.1	13	11.50%	0.0001	3.8
Carbohydrate metabolism	14	4.50%	0.0053	2.3	2	1.80%	0.6720	0.9
Sulfur metabolism	5	1.60%	0.0059	4.6	0			
Other metabolism	13	4.10%	0.0159	2.0	6	5.30%	0.0319	2.6
Cell adhesion	12	3.80%	0.0528	1.7	6	5.30%	0.0424	2.5
Others	176	56.10%			53	24.80%		

OR, odds ratio.

### Up- and downregulation of transcription factors

**Figure 4:**
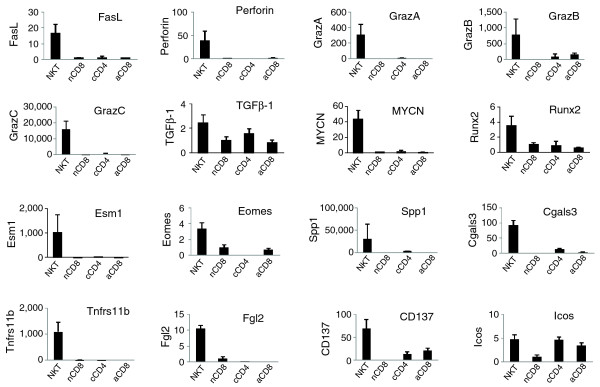
RT-PCR analysis of selected genes in four cell groups. Quantitative RT-PCR was performed in duplicate using cDNA (equivalent of 10 ng total RNA) and already-developed TaqMan gene expression assays (Applied Biosystems) on the ABI 7900 HT Fast Real-Time PCR System. Data were normalized based on 18srRNA and GAPDH expression. The mean expression level for naïve CD8^+^ T cells was artificially scaled to one for each tested gene. Data are presented as mean ± standard deviation.

The expression of a large number of transcription factors (TFs) was changed in the CD8^+ ^and CD4^+ ^T cells cultured using the expansion protocol (Table 3). Most of the differentially expressed TF genes were upregulated, while a small number were downregulated in the cultured cells. The expression patterns of the TF genes share some similarity but also have significant differences in the cultured CD8^+^, cultured CD4^+^, activated CD8^+ ^T cells and naïve CD8^+ ^T cells. Many of the TF genes still have unknown biological functions and their roles in T cells have not been investigated. However, several TF factors are known to be critical for the immune system and may play a role in gaining suppressive function for the cultured CD8^+ ^T cells. The V-*myc myelocytomatosis viral related oncogene, neuroblastoma derived *(*Mycn*) is essential to cell proliferation and differentiation [20]. This was the most upregulated TF gene (21-fold) in the cultured CD8^+ ^T cells but not in cultured CD4^+ ^(2-fold) or activated CD8^+ ^(1-fold) T cells (Table 3). RT-PCR analyses confirmed the expression differences observed with the microarray analysis (Figure 4). This may be a key gene for the cultured CD8^+ ^T cell phenotype. The Eomesodermin homolog (Eomes) is a T-box transcription factor that is highly homologous to T-bet. Eomes and T-bet may have cooperative or redundant functions in regulating the genes encoding IFN-γ and cytolitic molecules in CD8^+ ^T cells [21], and determine the fate of effector and memory CD8^+ ^T cells [22]. Furthermore, they are responsible for inducing enhanced expression of *Il2rb *(CD122) [22], a marker for some CD8^+ ^Treg cells [23]. *Eomes *was upregulated four-fold in the cultured CD8^+ ^T cells while it was downregulated five-fold in the cultured CD4^+ ^T cells and was unchanged by our activation protocol (Table 3). The upregulation of *Eomes *may be responsible for the increased expression of IFN-γ, perforin, granzymes, CD122 and other genes in cultured CD8^+ ^T cells. It could be a critical TF for the suppressive function of the cultured CD8^+ ^T cells. Runt related transcription factor 2 (Runx2) may be another critical transcription factor. *Runx2 *was highly upregulated in the cultured CD8^+ ^T cells (8.6-fold) and moderately upregulated in the cultured CD4^+ ^(3.5-fold) and activated CD8^+ ^(1.8-fold) T cells. Runx2 plays an important role in early T cell development [24]. Over-expression of *Runx2 *increases the proportion of single positive CD8^+ ^T cells [25]. Other potentially important TFs include Litaf, Jun (AP1), Zbtb32 (Rog), Zfp608 and Rnf13, which had higher expression levels in the cultured CD8^+ ^T cells than in the other three types of cells. The expression of Foxp3, which is an important TF for CD4^+ ^Treg cells, was not detectable by RT-PCR (data not shown) in the CD8^+ ^T cells cultured under this condition.

**Table 3:** Transcription factors differentially expressed in CD8^+ ^NKT-like cells

Symbol	Function	NKT/nCD8	CD4/nCD8	aCD8/nCD8	NKT/nCD8 (q)	CD4/nCD8 (q)	aCD8/nCD8 (q)
Nfil3	NF	19.7	22.3	4.3	4.0E-04	3.6E-04	3.4E-02
Mycn (Nmyc1)	TF	20.8	2.2	1.1	8.0E-05	2.7E-02	6.8E-01
Irf8 (Icsbp1)	TF	14.4	1.9	19.2	1.7E-04	3.2E-02	3.7E-03
Irf4	TF	4.7	24.7	18.2	1.6E-03	4.7E-05	6.2E-03
Litaf	TF	10.3	5.0	4.8	4.5E-05	1.4E-04	1.6E-02
Runx2	TF	8.6	3.5	1.8	2.2E-04	5.2E-02	3.4E-01
Pbx3	TF	5.8	6.2	1.6	4.1E-04	4.8E-04	3.6E-01
Jun (AP1)	TF	5.8	2.9	2.6	8.9E-04	8.8E-02	1.2E-01
Cgrrf1	TF	4.9	4.9	2.8	2.2E-04	2.7E-03	7.7E-02
Eomes	TF	4.0	0.2	0.6	9.7E-04	3.8E-04	4.5E-01
Atf4	TF	3.8	2.0	3.1	6.4E-03	3.1E-03	2.0E-02
Zbtb32 (Rog)	TF (ZF)	9.3	1.8	3.9	1.2E-03	3.9E-02	1.6E-02
Zdhhc2	TF (ZF)	4.9	2.6	1.5	9.3E-04	1.5E-03	4.6E-01
Zfp313	TF (ZF)	4.3	2.2	1.2	1.9E-02	6.6E-03	6.8E-01
Zfp608	TF (ZF)	3.9	1.2	1.7	4.2E-04	1.1E-01	1.3E-01
Rnf128	TF (RF)	6.2	10.4	1.0	5.8E-04	7.8E-04	8.0E-01
Rnf13	TF (RF)	4.0	1.7	1.2	8.5E-04	7.3E-02	7.0E-01
Socs2	Suppressor	48.6	104.0	27.8	3.5E-05	3.3E-05	3.9E-03
Cish (Socs)	Suppressor	8.8	9.5	4.9	1.2E-04	5.2E-05	1.3E-02
							
Tcf7	TF	0.012	0.061	0.274	5.7E-07	1.4E-03	7.7E-02
Klf3	TF (KR)	0.012	0.014	0.034	9.4E-08	5.9E-06	8.1E-03
Klf2	TF (KR)	0.04	0.03	0.01	4.3E-04	5.5E-04	2.7E-03
Klf1	TF (KR)	0.05	0.05	0.06	3.5E-05	1.7E-04	1.0E-02
Rkhd3	TF (RF)	0.15	0.17	0.16	1.1E-04	1.7E-04	9.1E-03
Bcl11a	TF (ZF)	0.15	0.16	0.15	2.4E-04	1.1E-03	2.3E-02
Zbtb20	TF (ZF)	0.20	0.15	0.16	1.2E-04	3.2E-04	6.3E-03

NKT, CD8^+ ^NKT-like cells; nCD8, naïve CD8^+ ^T cells; cCD4, cultured CD4^+ ^T cells; aCD8, activated CD8^+ ^T cells. KR, Kruppel-like factor; NF, nuclear factor; RF, ring finger; TF, transcription factor; ZF, zinc finger. The table is split into two parts based on the expression ratio of NKT/nCD8.

### The cultured CD8^+ ^T cells are CD8^+ ^NKT-like cells

**Figure 5:**
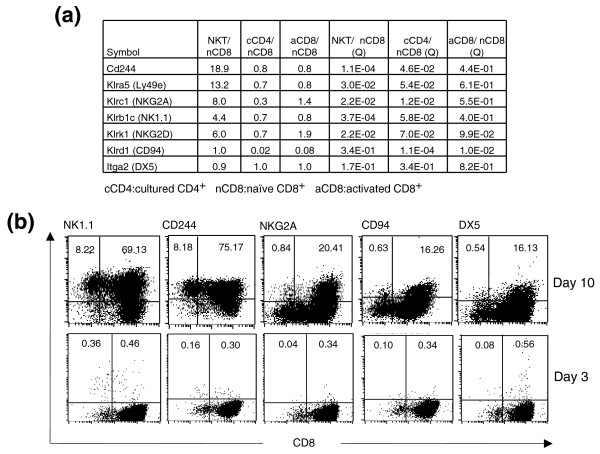
Expression of NK cell markers. **(a) **Summary of microarray data for NK cell markers. Ratios of expression values and FDR (q) values are presented. **(b) **NK cell marker expression on the surface of cultured CD8^+ ^NKT-like cells.

Several genes encoding surface markers on NK cells were highly upregulated in the cultured CD8^+ ^T cells (19-fold for CD244, 13-fold for Ly49e, 4.4-fold for NK1.1, 8.0-fold for NKG2A and 6-fold for NKG2D; Figure 5a) but not in the cultured CD4^+ ^or activated CD8^+ ^T cells. To confirm these findings, FACS analysis was carried out for a number of surface markers. As already mentioned, these cultured cells remained positive for CD8 (~99%) and negative for CD4 (Figure 1). They were activated T cells as indicated by the high expression levels of CD25 and CD69 as well as the low expression level of CD62L (Figure 1). Consistent with the low frequency of NKT cells among naïve CD8^+ ^T cells, <1% of the CD8^+ ^T cells were positive for these markers after three days of culture (Figure 5b), while the majority of the cells became positive for NK1.1 and CD244 after about 10 days of culture. The percentages of cells positive for the NK markers may vary from culture to culture. By day 10-13, 75-95% of the cells were normally positive for NK1.1 and CD244. NKG2A was upregulated by 8-fold in the cultured CD8^+ ^T cells according to the microarray data (Figure 5a) and 25-30% of the cultured CD8^+ ^T cells stained positive for NKG2A. Although CD94 and DX5 were not upregulated in the cultured CD8^+^ NKT-like cells according to the microarray data (Figure 5a), FACS analyses indicated that 15-30% of the cultured CD8^+ ^T cells were positive for these NK markers. It is unclear if these discrepancies are due to an imperfect correlation between gene and protein expression. Since the vast majority of the cultured CD8^+ ^T cells expressed NK markers, the cultured CD8^+ ^T cells had similar phenotypes to NKT cells, which are defined as cells expressing both T cell and NK cell markers. Furthermore, these cells were negative for the α-galactosylceramide-loaded Cd1d tetramer (data not shown), suggesting that they were not Cd1d-restricted *i*NKT cells. It is unclear at this time what the source of these cultured CD8^+ ^NKT-like cells was. As the CD8^+ ^NKT-like cell precursors in the total CD8^+ ^T cell pool were very rare, we believe that the cultured CD8^+ ^NKT-like cells were probably expanded from the conventional CD8^+ ^T cells, which acquired NK markers during the expansion. According to a recent classification of NKT cells [7], these cultured cells belong to the CD8^+ ^NKT-like category of NKT cells.

### Upregulation of secreted molecules with potential suppression functions

**Figure 6:**
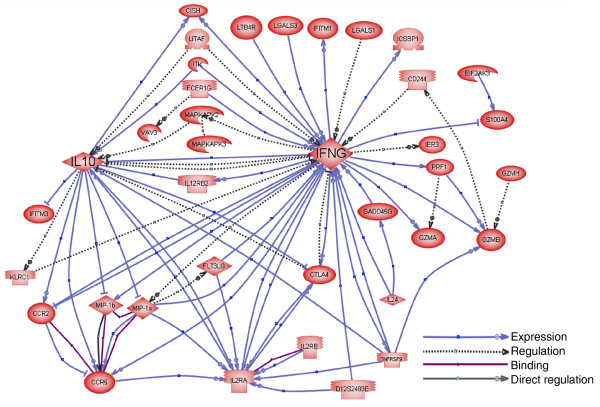
Molecular network for the highly upregulated immunity and defense genes. The network was created by extracting the direct interactions between these genes from the literature. Three types of relationship are shown in the pathway, binding, expression and regulation. Binding refers to physical interactions between molecules. Expression indicates that the regulator changes the protein level of the target by means of regulating its gene expression or protein stability. Regulation indicates that the regulator changes the activity of the target; the mechanism of the regulation is either unknown or has not been specified in the sentence describing the relationship. This network highlights the importance of two key nodes, IFN-γ and IL-10, which regulate many genes in this network. These genes are also critical for the immunosuppressive function of the CD8^+^ NKT-like cells.

**Figure 7:**
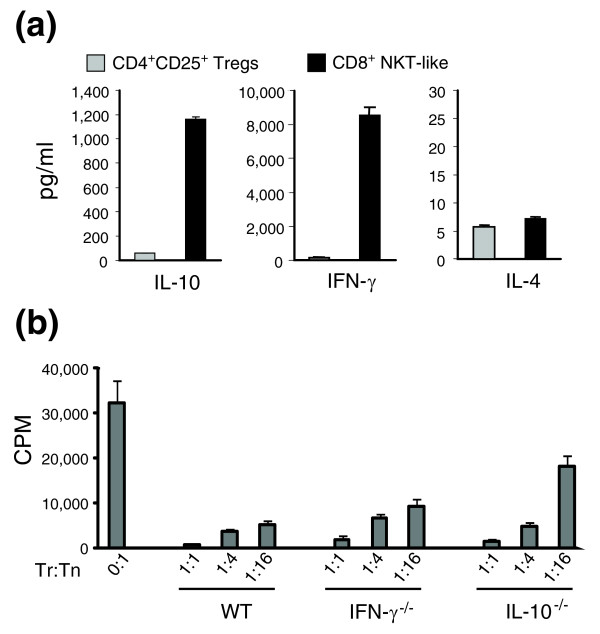
The role of IL-10 and IFN-γ in the generation and function of the CD8^+ ^NKT-like cells. **(a) **Cytokine levels in the cell culture media. Cultured CD8^+ ^NKT-like cells and freshly isolated CD4^+^CD25^+ ^Treg cells were stimulated with anti-CD3 (1.5 μg/ml) and splenic APCs. At 72 h of culturing, the culture supernatant was saved and used for measuring IL-10, IL-4 and IFN-γ using ELISA. Results are representative of two independent experiments. **(b) **Suppression activity of CD8^+ ^NKT-like cells cultured from IFN-γ^-/- ^and IL-10^-/- ^mice. CD8^+ ^NKT-like cells (Tr) cultured from knockout mice and wild-type B6 (WT) mice were co-cultured with naïve CD4^+^CD25^- ^responder T cells (Tn) at different Tr/Tn ratios in the presence of splenic APCs and anti-CD3. The cultures were pulsed with 1 μCi/well of [^3^H]thymidine at 72 h and proliferation (cpm) was measured by [^3^H]thymidine incorporation in the last 16 h. Results are expressed as the mean of triplicate cultures. ANOVA *p*-values are <0.0004 for IFN-γ^-/- ^and 0.001 for IL-10^-/- ^when compared to wild-type mice. Error bars are standard deviation.

Using the 318 genes that are upregulated by >5-fold in the cultured CD8^+ ^NKT-like cells, we established molecular networks to understand the functional relationships of the genes upregulated in the CD8^+ ^NKT-like cells. The largest network consists of genes involved in immunity and defense (Figure 6). This network highlights the importance of two central nodes: IL-10 and IFN-γ. Upon stimulation by anti-CD3/CD28 and IL-2, IL-10 and IFN-γ are highly upregulated (by 47- and 51-fold, respectively; Table 4). These proteins and pathways are known to influence the expression of many genes involved in immune responses, including those encoding the activation marker IL-2 receptor (Il2ra, or CD25), granzymes, the tumor necrosis factor (TNF) family genes, cytokines, chemokines and their receptors. Many of these genes are significantly upregulated in the CD8^+ ^NKT-like cells (Tables 4 and 5). To confirm the microarray data, we used ELISA to measure the levels of secreted cytokines in the culture medium of CD8^+ ^NKT-like cells and natural CD4^+^CD25^+ ^Treg cells stimulated by anti-CD3 and APC (Figure 7a). Consistent with the microarray data, the cultured CD8^+ ^NKT-like cells secreted more IL-10 and IFN-γ but a similar level of IL-4 when compared to fresh CD4^+^CD25^+ ^Treg cells (Figure 7a). The secretion of IFN-γ was particularly high in the CD8^+^ NKT-like cells. The expression of IFN-γ and lack of expression of IL-4 are also consistent with the observation on other NKT-like cells [7]. IL-10 and IFN-γ are immunosuppressive cytokines known to be involved in the suppressive function of CD4^+ ^Treg cells and may contribute to the suppressive function of the expanded CD8^+ ^NKT-like cells. Transforming growth factor (TGF)-β is another important immunosuppressive cytokine that might be important for the suppressive function of the CD8^+ ^NKT-like cells. Our microarray and RT-PCR data (Figure 4) indicate that the TGF-β mRNA level was about two-fold higher in CD8^+ ^NKT-like cells compared to naïve CD8^+ ^cells. As TGF-β cannot be accurately measured from serum-containing culture medium, we performed blocking experiments using an anti-TGF-β antibody to assess the role of TGF-β. Our results (Figure S1 in Additional data file 1) indicate that TGF-β blockade cannot block the suppression function of the CD8^+ ^NKT-like cells.

**Table 4:** Expression of genes encoding secreted molecules with potential suppressive function

Symbol	Function	NKT/nCD8	CD4/nCD8	aCD8/nCD8	NKT/nCD8 (q)	CD4/nCD8 (q)	aCD8/nCD8 (q)
Spp1	Suppression	251.4	203.8	7.4	9.9E-06	2.6E-06	1.2E-02
Lgals3 (Gal3)	Suppression	87.0	38.5	3.1	2.6E-07	1.7E-04	4.9E-02
Esm1	Suppression	74.5	2.8	1.2	4.2E-04	2.2E-02	5.8E-01
Fgl2	Suppression	32.7	0.9	0.9	9.2E-05	3.0E-01	6.3E-01
Tnfrsf11b (Opg)	Suppression	29.0	1.1	1.1	2.4E-04	3.0E-01	7.0E-01
Lgals1 (Gal1)	Suppression	27.1	22.3	6.0	1.4E-04	7.8E-04	6.1E-02
Gzmd	Killing	834.8	25.8	1.1	2.1E-07	1.0E-02	6.6E-01
Gzme	Killing	524.9	27.3	0.9	2.3E-06	6.8E-03	6.4E-01
Gzmc	Killing	446.4	25.7	2.1	2.7E-07	3.3E-02	1.8E-01
Gzmg	Killing	328.8	5.5	1.0	1.2E-05	1.8E-02	8.0E-01
Gzmb	Killing	104.6	60.2	85.6	2.2E-06	2.4E-04	2.8E-03
Gzmf	Killing	63.5	1.8	1.3	6.9E-04	9.4E-03	4.1E-01
Gzma	Killing	61.5	17.1	0.4	1.2E-05	6.8E-03	1.2E-01
Prf1	Killing	29.0	1.0	1.7	4.4E-04	4.1E-01	2.8E-01
Gzmk	Killing	23.3	0.5	0.6	1.5E-04	1.7E-02	2.7E-01
Ifng	Cytokine	51.2	50.3	258.7	3.3E-03	1.5E-02	8.2E-04
Il10	Cytokine	47.2	122.8	2.1	1.8E-03	1.6E-05	4.0E-02
Il24	Cytokine	5.5	33.0	1.0	5.1E-02	3.5E-04	8.1E-01
Lta	Cytokine	3.9	5.1	27.8	3.1E-03	2.2E-04	2.7E-03
Ccl3	Chemokine	86.2	162.4	177.9	3.4E-04	1.2E-03	1.3E-03
Ccl4	Chemokine	25.5	20.8	18.7	6.6E-04	3.5E-03	4.1E-03
Ccl9	Chemokine	11.3	53.7	4.4	1.5E-02	5.2E-04	4.5E-02
Cklfsf7	Chemokine	4.7	1.2	0.9	3.4E-03	1.8E-01	7.1E-01

NKT, CD8^+ ^NKT-like cells; nCD8, naïve CD8^+ ^T cells; cCD4, cultured CD4^+ ^T cells; aCD8, activated CD8^+ ^T cells.

**Table 5:** Gene expression levels for genes encoding critical surface markers

Symbol	Function	NKT/nCD8	CD4/nCD8	aCD8/nCD8	NKT/nCD8 (q)	CD4/nCD8 (q)	aCD8/nCD8 (q)
Ifitm1	Suppression	89.9	7.1	1.5	2.8E-07	2.2E-03	2.4E-01
Lilrb4 (ILT3)	Suppression	50.3	43.8	7.6	3.6E-04	4.0E-05	5.6E-03
Ifitm2	Suppression	45.6	11.4	1.6	6.0E-07	2.1E-03	2.8E-01
Havcr2 (Tim3)	Suppression	36.5	10.4	0.7	1.6E-04	2.0E-03	2.4E-01
Tnfrsf9 (4-1BB)	Suppression	28.2	12.5	12.8	2.4E-04	3.2E-03	1.1E-02
Tnfsf6 (FASL)	Suppression	24.4	4.3	3.2	1.2E-05	6.1E-02	4.5E-02
Ifitm3	Suppression	24.3	15.6	0.8	2.5E-05	1.4E-03	6.6E-01
Ctla4	Suppression	5.3	18.7	21.5	2.8E-03	4.8E-03	2.7E-03
Tnfrsf18 (GITR)	Suppression	4.8	11.1	4.4	2.7E-03	4.3E-05	1.7E-02
Pdcd1lg2	Suppression	4.6	7.3	3.3	5.4E-04	2.5E-04	3.0E-02
Icos	Suppression	4.6	4.0	3.6	2.8E-03	3.5E-03	3.2E-02
Tnfsf11 (RANKL)	Suppression	3.9	8.1	27.0	7.6E-03	2.4E-03	3.4E-03
Tnfsf10 (TRAIL)	Suppression	3.1	3.2	1.8	2.7E-03	9.5E-03	6.6E-02
Tnfrsf4 (OX40)	Suppression	3.0	23.8	11.3	1.6E-02	7.6E-06	5.7E-03
P2ry14 (Gpr105)	Receptor	65.4	6.9	1.1	2.1E-05	2.3E-03	6.8E-01
Fcer1g	Receptor	12.7	0.7	0.7	7.4E-04	1.4E-02	2.2E-01
Ptger3	Receptor	12.0	1.2	1.1	3.5E-04	2.3E-01	6.9E-01
Il12rb1	Receptor	10.6	11.3	4.0	8.9E-04	2.7E-05	1.8E-02
Ltb4r1	Receptor	8.8	1.2	0.9	2.9E-03	1.8E-01	7.0E-01
Gabarapl1	Receptor	8.7	5.3	3.4	2.4E-04	1.8E-03	1.4E-01
Ly6a	Receptor	7.3	11.3	3.0	3.5E-04	1.7E-04	5.3E-02
Tcrg	Receptor	6.3	0.2	0.3	1.9E-03	1.4E-03	6.9E-02
Il12rb2	Receptor	5.8	18.2	6.2	1.1E-04	1.6E-04	1.0E-02
Pilrb	Receptor	5.8	0.9	0.8	1.1E-03	1.5E-01	5.2E-01
Tmem2	Receptor	5.8	4.6	2.0	5.4E-03	8.5E-04	1.1E-01
Gpr171	Receptor	4.6	7.4	2.9	8.8E-03	1.3E-03	1.2E-01
Gpr34	Receptor	4.5	0.6	0.7	2.7E-02	1.1E-02	3.6E-01
Gpr160	Receptor	3.7	1.1	1.4	4.8E-03	3.0E-01	3.5E-01
Oprm1	Receptor	0.20	0.08	0.49	1.6E-03	3.6E-04	1.1E-01
Tlr1	Receptor	0.12	0.11	0.19	1.3E-04	3.5E-04	2.2E-02
Trat1	Receptor	0.12	0.15	0.25	1.3E-04	2.2E-03	4.3E-02
Edg1	Receptor	0.10	0.17	0.13	2.9E-04	1.0E-02	3.0E-02
Il2ra (CD25)	CR	48.4	66.9	28.1	1.7E-05	1.2E-05	2.7E-03
Il2rb (CD122)	CR	7.1	4.3	1.9	1.4E-03	1.4E-03	2.7E-01
Il7r	CR	0.12	0.17	0.05	3.4E-04	1.0E-04	4.9E-03
Il6ra	CR	0.11	0.26	0.16	6.3E-06	1.0E-02	9.2E-03
Il6st	CR	0.08	0.11	0.16	2.7E-07	3.7E-05	5.3E-03
Sema6d	Costimulation	6.1	0.9	1.4	2.1E-02	3.2E-01	2.5E-01
Pdcd1	Costimulation	4.5	4.0	4.6	8.3E-03	5.2E-03	1.7E-02
Ptger2	Costimulation	4.5	18.3	5.3	1.1E-04	6.8E-05	1.5E-02
Cd28	Costimulation	4.0	6.3	2.2	2.0E-03	2.6E-04	1.1E-01
Cd80	Costimulation	3.6	2.1	1.3	7.8E-03	5.3E-03	4.7E-01
Cd24a	Costimulation	0.03	0.82	0.78	4.2E-04	2.8E-01	6.4E-01
Ccr5	CCR	18.3	3.9	1.6	1.1E-05	4.3E-04	1.4E-01
Ccr2	CCR	15.7	0.7	0.5	2.4E-04	6.1E-02	4.0E-02
Cxcr6	CCR	5.0	1.9	0.1	5.5E-03	1.9E-01	3.6E-02
Ccr7	CCR	0.10	0.39	1.43	1.1E-03	6.2E-02	7.0E-01
Cxcr4	CCR	0.06	0.46	0.10	6.3E-06	2.1E-02	9.2E-03
Ccr9	CCR	0.03	0.03	0.04	3.1E-07	7.6E-06	2.7E-03
Adam8	Adhesion	16.9	6.1	0.8	3.9E-04	4.4E-03	4.5E-01
Tjp1	Adhesion	14.6	0.4	0.4	2.9E-05	6.7E-03	5.0E-02
Emp1	Adhesion	14.4	13.7	1.3	1.2E-03	2.2E-03	6.3E-01
Emilin2	Adhesion	13.6	0.8	1.1	2.4E-04	2.0E-01	7.6E-01
Itgav	Adhesion	10.6	3.6	2.3	3.6E-05	1.0E-03	8.1E-02
Nov	Adhesion	10.6	1.0	1.1	1.4E-02	4.3E-01	7.5E-01
Alcam	Adhesion	8.2	18.3	8.5	3.0E-04	2.9E-04	1.9E-02
Cdh1	Adhesion	6.9	0.8	0.9	3.3E-03	8.6E-02	6.2E-01
Adam9	Adhesion	6.9	7.1	2.3	4.0E-04	1.2E-03	2.6E-01
Tjp2	Adhesion	5.7	4.7	2.0	1.8E-04	9.1E-05	1.0E-01
Dsc2	Adhesion	5.0	0.9	0.9	5.8E-02	2.0E-01	7.1E-01
Itga6	Adhesion	0.14	0.12	0.48	4.2E-04	1.6E-04	1.5E-01
Itgae	Adhesion	0.08	0.10	0.09	3.8E-06	7.1E-05	4.2E-03
Sell (CD62L)	Adhesion	0.04	0.23	0.19	2.4E-04	3.6E-04	1.4E-02

NKT, CD8^+ ^NKT-like cells; nCD8, naïve CD8^+ ^T cells; cCD4, cultured CD4^+ ^T cells; aCD8, activated CD8^+ ^T cells. CCR, chemokine receptor; CR, cytokine receptor.

A number of other secreted molecules were also highly upregulated in the CD8^+ ^NKT-like cells based on the microarray data (Table 4). Many of these secreted molecules are known to have immunosuppressive function or potentially contribute to the suppression function. Perforin (*Prf1*) and granzymes are among the most noticeable. Perforin and granzyme expression is regulated by IFN-γ (Figure 6). Both natural and adaptive CD4^+^CD25^+ ^Treg cells in human display perforin-dependent cytotoxicity against autologous target cells, suggesting that the perforin/granzyme pathway is one of the mechanisms that Treg cells can use to control immune responses [26]. *Prf1 *was upregulated by 29-fold in the CD8^+^ NKT-like cells (with potent suppression activity; Table 4), but unchanged in cultured CD4^+ ^cells (with weak suppression activity) and activated CD8^+ ^T cells (without suppression activity). Several granzymes were highly upregulated (834-, 535-, 446-, 329-, 105-, 63-, 61- and 23-fold for granzymes D, E, C, G, B, F, K and A, respectively). These molecules were generally upregulated to a much lesser degree in the cultured CD4^+ ^T cells and were unchanged in activated CD8^+ ^T cells (except *Gzmk*). The large expression differences for *Prf1 *and selected granzymes were confirmed using RT-PCR (Figure 4).

Several secreted molecules can potentially be implicated in the immunosuppressive function. The most noticeable include Esm1, Spp1, Fgl2, Tnfrsf11b, Lgals3, Lgals1, and IL-24 (Table 4). *Esm1 *(endothelial cell-specific molecule 1) was upregulated in the CD8^+ ^NKT-like cells by 75-fold and was only slightly increased in the cultured CD4^+ ^T cells (2.8-fold) and was unchanged in the activated CD8^+ ^T cells. The expression pattern was confirmed by RT-PCR (Figure 4). Esm1 is a proteoglycan mainly secreted by endothelial cells under the control of inflammatory cytokine. It binds to LFA-1 integrin on the surface of lymphocytes and monocytes [27] and therefore inhibits the binding of intercellular adhesion molecules (ICAMs) to LFA-1 and influences leukocyte adhesion and activation. Spp1 (secreted phosphoprotein 1) is better known as osteopondin. In addition to its well known function in bone formation, it functions as a cytokine and chemokine to regulate cell-cell and cell-tissue interaction. Much less well known is its function in suppressing T cells and activating B cells [28]. Osteopondin is believed to be the most abundant protein secreted by activated T cells, which is consistent with our microarray data (7.4-fold higher expression in activated CD8^+ ^T cells versus naïve T cells). Osteopondin was upregulated by 252-fold in the CD8^+ ^NKT-like cells and 204-fold in the cultured CD4^+ ^T cells based on the microarray data (Table 4). Based on the RT-PCR data, Osteopondin (Spp1) was greatly increased (by 25,000-fold) in the CD8^+^ NKT-like cells compared to the naïve CD8^+^ T cells (Figure 4). It is possible that Osteopondin contributed to the suppression activity of both the CD8^+ ^and CD4^+ ^T cells cultured using our protocol. Tnfrsf11b, also known as Osteoprotegerin (Opg), is a member of the TNF receptor superfamily. Opg is a decoy receptor of RANKL and inhibits the binding of RANKL (Receptor activator for nuclear factor κB ligand) to its receptor RANK. Opg is secreted as a disulfide-linked homodimer [29]. Opg can inhibit the inflammatory effect of RANKL secreted by activated T cells [30,31] and RANKL blockade can significantly prolong heart allograft survival [32]. Opg was upregulated by 29-fold in the CD8^+^ NKT-like cells and unchanged in the cultured CD4^+ ^and activated CD8^+ ^T cells. The expression changes were also confirmed by RT-PCR (Figure 4). Fgl2 (Fibrinogen-like protein 2) is a member of the fibrinogen-related protein superfamily. In addition to its well established role in triggering thrombosis, it is known to be secreted by T cells under the control of IFN-γ [33]. Fgl2 has been shown to exhibit immunomodulatory properties capable of inhibiting dendritic cells (DC) maturation and T cell proliferation stimulated by alloantigens or anti-CD3/anti-CD28 antibodies in a dose-dependent manner [34]. *Fgl2 *was upregulated by 33-fold in the CD8^+ ^NKT-like cells but was unchanged in the cultured CD4^+ ^and activated CD8^+ ^T cells. Thus, Fgl2 could be a critical factor for the suppression mechanism of the CD8^+ ^NKT-like cells. Lgals3 and Lgals1, also known as Galectin (Gal)-3 and Gal1, are members of the beta-galactoside-binding gene family. They are multifunctional proteins implicated in a variety of biological functions, including tumor cell adhesion, proliferation, differentiation, angiogenesis, cancer progression and metastasis. It was recently shown that Gal3 secreted by tumor cells induces T cell apoptosis [35]. The expression of Gal3 has been positively correlated with the level of apoptosis of tumor-associated lymphocytes [36]. Treatment with the *Gal3 *gene is also beneficial against asthma in mice [37]. Finally, IL-24 is a member of the IL-10 family of cytokines [38]. Over-expression of IL-24 induces apoptosis in cancer cells [39]. Therefore, IL-24 appears to be an immunosuppressive cytokine.

### Cultured CD8^+ ^NKT-like cells upregulate many suppressive surface markers

A large number of surface molecules were highly upregulated in the CD8^+ ^NKT-like cells, while a few surface molecules were down regulated (Table 5). Many of the upregulated molecules have been implicated in immunosuppressive function. Most notably, many of the genes are related to IFN-γ and some belong to the TNF family receptors and ligands. The expression patterns for these genes are clearly different among the cells cultured under different conditions or different cell types cultured under the same condition. The overall pattern seems to correlate well with their cellular functions. The genes already implicated in suppressive function or having suppressive potential were highly upregulated in the CD8^+ ^NKT-like cells, which have potent suppression activity, while these genes were only moderately upregulated or unchanged in the cultured CD4^+ ^T cells and activated CD8^+ ^T cells, which have only weak or no suppression activity.

Ifitm1 (Interferon induced transmembrane protein 1) is the most upregulated surface molecule in the CD8^+ ^NKT-like cells (90-fold increase compared to naïve CD8^+ ^T cells; Table 5). This gene is not upregulated by the conventional activation protocol and upregulated to a much lesser degree in the cultured CD4^+ ^T cells. Ifitm1 has been shown to be a key molecule in the anti-proliferative function of IFN-γ [40]. Two other interferon-induced transmembrane genes (*Ifitm2 *and *Ifitm3*) were also highly upregulated in the CD8^+ ^NKT-like cells (45- and 24-fold, respectively). It is highly likely that these proteins are involved in the suppressive function of the CD8^+ ^NKT-like cells.

Lilrb4 (Leukocyte immunoglobulin-like receptor, subfamily B, member 4) is a member of the leukocyte immunoglobulin-like receptor (LIR) family. The encoded protein belongs to the subfamily B class of LIR receptors with a transmembrane domain, extracellular immunoglobulin domains, and cytoplasmic immunoreceptor tyrosine-based inhibitory motifs. The receptor expressed on immune cells binds to MHC class I molecules on antigen-presenting cells and transduces a negative signal that inhibits stimulation of an immune response. The receptor can also function in antigen capture and presentation. It may be involved in controlling inflammatory responses and cytotoxicity to help focus the immune response and limit autoreactivity. This gene was highly upregulated in both the CD8^+ ^NKT-like cells and cultured CD4^+ ^T cells.

Havcr2 (Hepatitis A virus cellular receptor 2), more commonly known as Tim3, was upregulated by 36-fold in the CD8^+ ^NKT-like cells and 5-fold in the cultured CD4^+ ^T cells compared to naïve CD8^+ ^T cells. Tim3^-/- ^mice have exacerbated diabetes due partly to a defect in CD4^+^CD25^+ ^Treg cell function [41]. Therefore, Tim3 may be important for CD8^+ ^NKT-like cell suppression function. Tnfrsf9, also known as 4-1BB and CD137, was highly upregulated in the CD8^+ ^NKT-like cells (30-fold) and only slightly upregulated in the cultured CD4^+ ^T cells (5-fold). 4-1BB is a costimulatory molecule that may be very important for Treg cell function. 4-1BB-primed CD8^+ ^T cells possess suppressive function [42] and an agonist monoclonal antibody specific for 4-1BB can mitigate autoimmunity [43-47]. 4-1BB^-/- ^mice exhibit enhanced T cell proliferation [48]. GITR (Tnfrsf18) is an important molecule for CD4^+ ^Treg cell function. GITR was upregulated 5-fold in the CD8^+ ^NKT-like cells and 10-fold in the cultured CD4^+ ^T cells. Clearly, GITR could contribute to the suppressive function of both CD4^+ ^and CD8^+ ^Treg cells. Other upregulated costimulatory molecules, such as Pdcd1 (5.6-fold), PDL2 (4.6-fold) and Icos (4.3-fold), Ctla4 (5-fold) and CD28 (3.9-fold), may be good candidate molecules involved in the suppressive function. CD24a is one of the few costimulatory molecules that was downregulated in the CD8^+ ^NKT-like cells (0.03-fold) but unchanged in the cultured CD4^+ ^T cells (1.2-fold).

Genes for several TNF family proteins, such as Fas-L, RANKL, TRAIL and OX40, were all upregulated in the CD8^+ ^NKT-like cells and the cultured CD4^+ ^T cells. All these molecules could be implicated in the suppression function. Fas-L expression was 25-fold higher in the CD8^+^ NKT-like cells compared to naïve CD8^+ ^T cells while CD4^+ ^T cells cultured in the same condition did not upregulate Fas-L. Fas/Fas-L is one of the two pathways of lymphocyte-mediated cell killing [49].

Several lymphocyte receptors were upregulated - Gpr105 (65-fold), Ptger3 (13.1-fold), Ptger2 (4.3-fold), Fcer1g (13.7-fold) - and a few other receptors were downregulated by the expansion protocol (Table 4). Prostaglandin E receptor 3 (subtype EP3; Ptger3) may have pro-inflammatory or anti-inflammatory properties depending on the physiological condition [50]. EP2, EP3 and EP4 receptors have been shown to be important for the immunosuppressive function of PGE2 [51]. Cytokine and chemokine receptors were up- or downregulated by the expansion protocol (Table 5). Notably, Il2ra (CD25) was highly upregulated in the CD8^+ ^NKT-like and cultured CD4^+ ^T cells as well as activated CD8^+ ^T cells. These expected results were confirmed by FACS analysis (Figure 1). Ccr5 and Ccr2 were highly upregulated in the CD8^+ ^NKT-like cells but unchanged or slightly upregulated in the cultured CD4^+ ^T cells and activated CD8^+ ^T cells. Ccr5 has been shown to be important for the suppression function of CD4^+^CD25^+ ^T cells [52]. CCR5 and several other CCR members (CCR2) were highly upregulated in the CD8^+ ^NKT-like cells (Table 3) and they should be excellent candidate molecules for further studies.

A large number of adhesion molecules were highly upregulated in the CD8^+ ^NKT-like cells and only slightly upregulated/unchanged or even decreased in the cultured CD4^+ ^T cells (Table 5). Several of these genes (*Tjp1*, *Emilin2*, *Nov*) were confirmed by RT-PCR (Figure 4) and Sell (CD62L) was confirmed by FACS analysis (Figure 1).

### IL-10 and IFN-γ are two key pathways for the conversion and function of the CD8^+ ^NKT-like cells

Immunosuppressive cytokines can enhance the suppressive activity of Treg cells. For example, naturally occurring CD4^+^CD25^+ ^Treg cells use a combination of IL-10 and TGF-β to suppress immune responses [53-57]. As IL-10 and IFN-γ are two key nodes of the molecular network modified in the CD8^+^ NKT-like cells (Figure 6), we further tested their role in the function and generation of these cells using two different approaches. First, an anti-IL-10 antibody was used in the suppression assay to determine whether IL-10 neutralization could block or reduce the suppression function of the CD8^+ ^NKT-like cells. Blockade using anti-IL-10 could slightly reduce the suppression but could not completely block the suppressive function of the CD8^+ ^NKT-like cells (data not shown). Since the levels of IL-10 and IFN-γ secreted by the CD8^+ ^NKT-like cells was very high, antibody blocking may not have been efficient. Furthermore, the antibody blockade experiment evaluated only the role of these cytokines in the suppression function but did not allow us to assess the role of these molecules in the generation of the CD8^+ ^NKT-like cells. Therefore, we tested the potential roles of IL-10 and IFN-γ in CD8^+ ^NKT-like function/generation using IL-10^-/- ^and IFN-γ^-/- ^mice. CD8^+^ NKT-like cells can be cultured using naïve CD8^+ ^T cells from both knockout mice; however, at the later stages of culture, the viability of cells from IFN-γ^-/- ^mice is not as good as those from wild-type mice and IL-10^-/- ^mice. Addition of IFN-γ in the culture medium can improve the viability of the cultured cells (Figure S2 in Additional data file 1). Although the CD8^+ ^NKT-like cells cultured from both knockout mice had good suppressive activity at a high ratio (Tr/Tn = 1:1) of suppressor (CD8^+ ^NKT-like) to responder (naïve CD4^+ ^T cells), the reduction in suppressive activity for the CD8^+ ^NKT-like cells cultured with naïve CD8^+ ^T cells from both knockout mice became very clear at lower suppressor to responder T cell ratios (Figure 7b), which provides a more accurate estimate of the suppressive activity. At the 1:16 Tr/Tn ratio, the suppressive activity of CD8^+ ^NKT-like cells cultured from IL-10^-/- ^mice was reduced to 30-35% of that for CD8^+ ^NKT-like cells cultured from wild-type B6 mice (Figure 7b), suggesting that the vast majority of the suppressive activity of the CD8^+ ^NKT-like cells can be attributed to the IL-10 pathway. Similarly, almost 50% of the CD8^+ ^NKT-like suppression could be attributed to the IFN-γ pathway (Figure 7b). These results together suggest that IL-10 and IFN-γ play an important role in the *in vitro *generation and function of CD8^+ ^NKT-like cells.

## Discussion

Naïve CD8^+ ^T cells cultured with anti-CD3/anti-CD28-coated beads in the presence of a high concentration of IL-2 can generate CD8^+ ^NKT-like cells. This highly efficient culture system can produce large numbers of CD8^+ ^NKT-like cells. The cultured CD8^+ ^NKT-like cells can potently suppress the proliferation of responder T cells. In this study, we extensively characterized the molecular mechanisms underlying the suppression function of the cultured CD8^+ ^NKT-like cells using a variety of techniques. We first compared the gene expression profiles of four different cells with different phenotypes with regard to suppression function. The CD8^+ ^NKT-like cells cultured with the expansion protocol were highly potent suppressor cells (Figure 2). The suppressive activity of these NKT-like cells was actually much more potent than that of natural CD4^+^CD25^+ ^Treg cells, which are known to be highly suppressive. To identify candidate genes that might be related to the suppressive activity, the CD8^+ ^NKT-like cells with potent suppressive activity were compared to three different control cells: CD4^+ ^T cells (with very weak suppression) cultured under the same conditions as a control for culture condition; naïve CD8 T cells as the baseline expression level; and CD8^+ ^T cells activated using conventional activation protocols. The last two cell populations do not possess suppressive activity. This data set allows us to examine a number of different questions; however, we focus on the suppressive mechanism of the cultured CD8^+ ^NKT-like cells in this paper.

The number of genes modified by the expansion protocol in both CD8^+ ^and CD4^+ ^T cells was quite extensive. Approximately 3,000 genes were changed by the culture. While some genes were downregulated, most genes were upregulated by the expansion protocol. In contrast, the number of genes significantly changed by the activation protocol was much less. Another surprise with this dataset is the extent of the gene expression changes in a large number of genes. Several dozens of genes were changed by 40- to 800-fold. Whereas the exact extent of gene expression changes may not be accurately measured for all genes, confirmatory studies using a variety of techniques did provide evidence that many of the genes showed large differences.

If one focuses on the upregulated genes with a false discovery rate (FDR) (q) of <0.01 and >5-fold difference between CD8^+ ^NKT-like cells and naïve CD8^+ ^T cells, a large number of genes encode proteins that have already been implicated in immune suppression or have functions consistent with immune suppression, the critical phenotype for these cultured CD8^+ ^NKT-like cells. The proteins with immunosuppressive properties include both surface molecules and soluble/secreted molecules. Many of the surface molecules belong to the TNF family and interferon-regulated proteins (Table 5). Many of these molecules were highly upregulated in the CD8^+ ^NKT-like cells but not upregulated in the cultured CD4^+ ^T cells or activated CD8^+ ^T cells. Which of these molecules are involved in suppressing T cell proliferation by the CD8^+ ^NKT-like cells remains to be investigated in future studies. It is likely that these molecules may work cooperatively to confer suppressive function. Multiple molecules may have to be blocked to demonstrate the suppression function of these molecules.

Furthermore, the CD8^+ ^NKT-like cells upregulated a large number of genes encoding secreted proteins that are known, or have the potential, to be implicated in the suppression function. These molecules include Fas-L, perforin, granzymes, Spp1, Lgals3, Lgal1 and others. Again, any of these molecules may confer some suppression function and the potent suppressive function of the CD8^+ ^NKT-like cells may be related to more than one of these molecules. Our cell culture system provides an excellent model to further investigate the function of these molecules in immune suppression.

Pathway analysis of the expression data identified IL-10 and IFN-γ as two critical nodes linking many of the upregulated genes that may be implicated in immune suppression (Figure 6). Microarray and ELISA data both suggest that the CD8^+ ^NKT-like cells express high levels of IL-10 and IFN-γ. Previous studies suggested that these immunosuppressive cytokines could be directly involved in the immune suppressive function. The suppressive activities of some regulatory T cells like Tr1 cells have been attributed to their IL-10 production [58,59], while CD4^+^CD25^+ ^T cells produce less IL-10 and can suppress via IL-10-dependent or -independent mechanisms [54,56]. Some studies demonstrated production of both IL-10 and IFN-γ by CD4^+ ^Treg cells [60]. IFN-γ has also been reported to be a suppressive cytokine secreted by T cells [42,61]. In certain models, IFN-γ is identified as part of a suppressive pathway [62]. Thus, despite its pro-inflammatory functions, IFN-γ may contribute to the regulation of T-cell responses [63,64] as shown in IFN-γ^-/- ^mice [65] and in graft versus host responses [66,67]. Our studies using IL-10^-/- ^and IFN-γ^-/- ^mice clearly indicate that IL-10 and IFN-γ both play a role in the generation and/or function of CD8^+ ^NKT-like cells. However, IL-10 or IFN-γ alone cannot completely explain the suppressive function of the cultured CD8^+ ^NKT-like cells. It will be interesting to find out whether IL-10^-/-^-IFN-γ^-/- ^double knockout can completely abolish the conversion and/or function of CD8^+ ^NKT-like cells. It is possible that other compensatory pathways may exist for the production and/or function of CD8^+ ^NKT-like cells. In addition, it will be interesting to identify the signaling pathways upstream of IL-10 and IFN-γ. In both regards, further investigation of the large number of differentially expressed transcription factors may provide important clues.

## Conclusion

This study demonstrates that CD8^+ ^NKT-like cells generated from *in vitro *culture possess potent immune suppressive activity. Gene and protein expression studies using a variety of techniques, including microarray analysis, RT-PCR, ELISA and FACS analyses as well as functional characterization using knockout mice, demonstrate the involvement of two key molecular pathways, IL-10 and IFN-γ, in the function of these potent suppressor T cells. Our culture system and the molecular information provide a valuable platform for the further dissection of the molecular and functional pathways implicated in the conversion and function of suppressor T cells.

## Materials and methods

### Mice

C57BL/6 (B6), B6.IL-10^-/- ^and B6.IFN-γ^-/- ^mice were purchased from Jackson Lab (Bar Harbor, ME, USA) and housed/bred under specific pathogen-free conditions at the Medical College of Georgia Animal Barrier Facility. This study was approved by the Medical College of Georgia IACUC committee.

### Cell sorting and flow cytometry

CD8^+ ^T cells were enriched from spleens by negative selection using an AutoMACS from Miltenyi Biotec (Auburn, CA, USA). The resulting CD8^+ ^cell purity was around 90-95%. High purity CD4^+ ^T cells were obtained by sorting splenic T cells using a Mo-Flow cytometer (Dako, Carpinteria, CA, USA). Flow cytometric analyses were performed on a FACScalibur™ flow cytometer with CELLQuest™ software (Becton Dickinson, Franklin Lakes, NJ, USA).

### *In vitro *culture of CD8^+ ^and CD4^+ ^T cells

AutoMACS-purified naïve CD8^+ ^or Mo-Flow sorted CD4^+ ^T cells at 10^6 ^cells/ml were cultured with anti-CD3 and anti-CD28 coupled to 4.5 mm paramagnetic Dynal beads (Invitrogen, Carlsbad, CA, USA) supplemented with 2,000 IU/ml rIL-2 (PeproTech, Rocky Hill, NJ, USA) in complete medium, which consisted of 10% heat-inactivated fetal bovine serum (Sigma-Aldrich, St. Louis, MO, USA), nonessential amino acids, 0.5 mM sodium pyruvate, 5 mM Hepes, 1 mM glutaMax I (all from Invitrogen, Carlsbad, CA, USA), and 55 μM mercaptoethanol (Sigma-Aldrich) in DMEM base. The cultures were monitored daily and maintained at 1-1.5 × 10^6 ^cells/ml by diluting with IL-2-supplemented complete medium for 8-13 days. At the end of the culture, the anti-CD3 and anti-CD28 beads were removed using a Dynal MPC-L magnet (Dynal Biotech), and the cells were routinely assayed for CD8, CD4, CD62L, and CD25 expression by flow cytometry and for *in vitro *suppression assays.

### *In vitro *suppression assays

Two different types of suppression assays were performed in this study. Most of the studies were performed using the CFSE system. Briefly, naïve CD4^+^CD25^- ^responder T cells were labeled with 2.5 μM CFSE. These labeled responder cells (1 × 10^5^) were cocultured at 37°C with different numbers of suppressor cells (CD8^+ ^NKT-like cells, cultured CD4^+ ^T cells or CD4^+^CD25^+ ^Treg cells) in the presence of 1.5 × 10^5 ^irradiated (2,000 rads) splenic APC (T cell depleted spleen cells) and 1.5 μg/ml anti-CD3 in a U-bottomed 96-well plate. After 72 h of culture, responder T cell proliferation was assessed by determining the dilution of CFSE using flow cytometry. The second type of suppression assay has identical culture conditions but the responder T cells were not labeled. The cultures were pulsed with 1 μCi/well [^3^H]thymidine for the last 16 h of 72 h culture and the level of proliferation was assessed by [^3^H]thymidine incorporation using scintillation counting after cell harvesting.

### Cytokine analysis by ELISA

Cells were cultured in 96-well plates with 1 × 10^5 ^CD4^+^CD25^- ^T cells/well, 1.5 × 10^5^/well irradiated splenic APC and 1.5 μg/ml anti-CD3 in the presence or absence of 1 × 10^5^/well CD8^+ ^NKT-like cells or fresh CD4^+^CD25^+ ^Treg cells. The level of IL-10, IFN-γ, IL-4 and IL-2 in the culture supernatant was determined by ELISA kits purchased from R&D Systems (Minneapolis, MN, USA).

### Microarray experiments

Total RNA was extracted from cultured or fresh T cells using the Qiashredder column and RNeasy Mini kit (Qiagen Inc., Valencia, CA, USA). All RNA extracted was analyzed for quantity and quality using the Agilent 2100 Bioanalyzer system (Agilent Technologies, Palo Alto, CA, USA). Gene expression profiling was performed using the mouse genome 430 2.0 chips (GeneChip™, Affymetrix, Santa Clara, CA, USA). An aliquot of 1 μg of total RNA was converted into double-stranded cDNA (ds-cDNA) by using SuperScript Choice System (Gibco BRL Life Technologies, Carlsbad, CA, USA) with an oligo-dT primer containing a T7 RNA polymerase promoter (Genset, San Diego, CA, USA). After second-strand synthesis, the reaction mixture was extracted with phenol-chloroform-isoamyl alcohol, and ds-cDNA was recovered by ethanol precipitation. *In vitro *transcription was performed on the above ds-cDNA using the Enzo RNA transcript Labeling kit. Biotin-labeled cRNA was purified by using an RNeasy affinity column (Qiagen), and fragmented randomly to sizes ranging from 35-200 bases by incubating at 94°C for 35 minutes. The hybridization solutions contained 100 mM MES [2-(N-morpholino)ethanesulfonic acid], 1 M Na^+^, 20 mM EDTA, and 0.01% Tween 20. The final concentration of fragmented cRNA was 0.05 μg/μl in hybridization solution. Target for hybridization was prepared by combining 40 μl of fragmented transcript with sonicated herring sperm DNA (0.1 mg/ml), bovine serum albumin and 5 nM control oligonucleotide in a buffer containing 1.0 M NaCl, 10 mM Tris.HCl (pH7.6), and 0.005% Triton X-100. Target was hybridized for 16 h at 45°C to a set of oligonuceotide arrays (Affymetrix). Arrays were then washed at 50°C with stringent solution, then again at 30°C with non-stringent washes. Arrays were then stained with streptavidin-phycoerythrin (Invitrogen). DNA chips were read at a resolution of 3 μm with a Hewlett-Packard GeneArray Scanner and were analyzed with the GENECHIP software (Affymetrix GCOS 1.1). Both the CEL and DAT files for each hybridization have been uploaded to our server running GeneTraffic v3.2 (Iobion Informatics LLC, La Jolla, CA, USA).

### Data analysis

Microarray data were first normalized using RMA [68] and normalized data were subsequently analyzed using the LIMMA [69] package in R. All groups were compared pairwise, and the resulting *p*-values were adjusted using the pFDR of Storey [70] and the qvalue package in R. We considered all probesets with a q-value ≤0.01 as being significant. The heatmap was constructed using the heatmap.2 function in R. Differentially expressed genes showing more than five-fold change between CD8^+ ^NKT-like and naïve CD8^+ ^T cells were annotated with respect to their involvement in biological processes and pathways using the PANTHER (Protein ANalysis THrough Evolutionary Relationships) classification system [71]. Of the 314 genes, biological processes for 257 were found by PANTHER. One-tailed *p*-values for enrichment of particular biological processes were obtained using the standard Fisher's exact test, to determine if the observed number of counts exceeded the expected counts. Gene lists from each significantly enriched (*p *< 0.05) biological process were further analyzed by pathway analysis. A network depicting the molecular interactions between the given set of genes was constructed using Pathway Studio Version 5.0 software and ResNet mammalian database (Ariadne Genomics, Rockville, MD, USA). ResNet is a very large database of molecular interactions and biological relationships extracted from the biomedical literature. The found interactions between the given set of genes were manually curated by reading sentences from which the relationship or interaction was derived. Only manually curated interactions were used for the final pathway visualization.

### RT-PCR analysis

An aliquot of total RNA (2 μg per sample) were arrayed in 96-well plates and then converted to cDNA using a High Capacity cDNA Reverse Transcription Kit (Applied Biosystems, Foster City, CA, USA) and a programmable thermal cycler (Applied Biosystems). The cDNA products were diluted and an aliquot of cDNA equivalent to 10 ng total RNA was used for quantitative RT-PCR performed using ready-to-use TaqMan gene expression assays from Applied Biosystems. 18srRNA and GAPDH were used as endogenous controls for normalizing RNA concentration. RT-PCR was performed in 384-well plates with the ABI 7900HT Fast Real-Time PCR System (Applied Biosystems). Standard thermal cycling conditions (10 minutes at 95°C, 40 cycles for 15 s at 95°C, 1 minute at 60°C) was used for all genes. All samples were analyzed on the same plate and each sample was analyzed in duplicate. Cycle threshold (C_T_) values for each test gene and 18SrRNA and GAPDH were obtained for each sample using the SDS2.3 and analyzed with RQ Manager 1.2 software (Applied Biosystems). Differences in C_T _values between a test gene and 18srRNA (ΔC_T_) for each sample were calculated and used for statistical analyses.

## Abbreviations

APC, antigen presenting cell; CFSE, carboxyfluorescein succinimidyl ester; ds-cDNA, double-stranded cDNA; ELISA, enzyme-linked immunosorbent assay; FACS, fluorescence-activated cell sorting; FDR, false discovery rate; Fgl, Fibrinogen-like protein; Gal, Galectin; Ifitm, Interferon induced transmembrane protein; IFN, interferon; IL, interleukin; MHC, major histocompatibility complex; NK, natural killer; NKT, natural killer T cell; Opg, Osteoprotegerin; RANK, Receptor activator for nuclear factor κB; RANKL, RANK ligand; TCR, T cell receptor; TF, transcription factor; TGF, transforming growth factor; Tn, naïve T; TNF, tumor necrosis factor; Treg, regulatory T.

## Authors' contributions

LZ performed most experiments and drafted the manuscript. HW carried out most RT-PCR analyses. XZ participated in the cellular studies. YJ performed RT-PCR analyses. QM participated in experimental design and data interpretation. RAM participated in data analysis and interpretation. NG and RP performed statistical analysis. J-XS participated in experimental design, data analysis and interpretation, writing and editing of the manuscript.

## Additional data files

The following additional data are available. Additional data file 1 contains two figures showing that anti-TGF-β cannot block the suppression function of CD8^+ ^NKT-like cells and that IFN-γ restores the viability of *in vitro *cultured CD8^+ ^NKT-like cells from IFN-γ^-/- ^mouse.

## Supplementary Material

Additional data file 1 Figure S1: anti-TGF-β cannot block the suppression function of CD8^+ ^NKT-like cells. Figure S2: IFN-γ restores the viability of *in vitro *cultured CD8^+ ^NKT-like cells from IFN-γ^-/- ^mouse. Click here for file
