# Supplementary material for: The IL-10 and IFN-γ pathways are essential to the potent immunosuppressive activity of cultured CD8+ NKT-like cells
Source: Genome Biol. 2008 Jul 29;9(7):R119. doi: 10.1186/gb-2008-9-7-r119 (PMC2530876; doi:10.1186/gb-2008-9-7-r119)
Supplement: Additional data file 1 — Figure S1: anti-TGF-β cannot block the suppression function of CD8+ NKT-like cells. Figure S2: IFN-γ restores the viability of in vitro cultured CD8+ NKT-like cells from IFN-γ-/- mouse. [file gb-2008-9-7-r119-S1.ppt]

## Slide 1
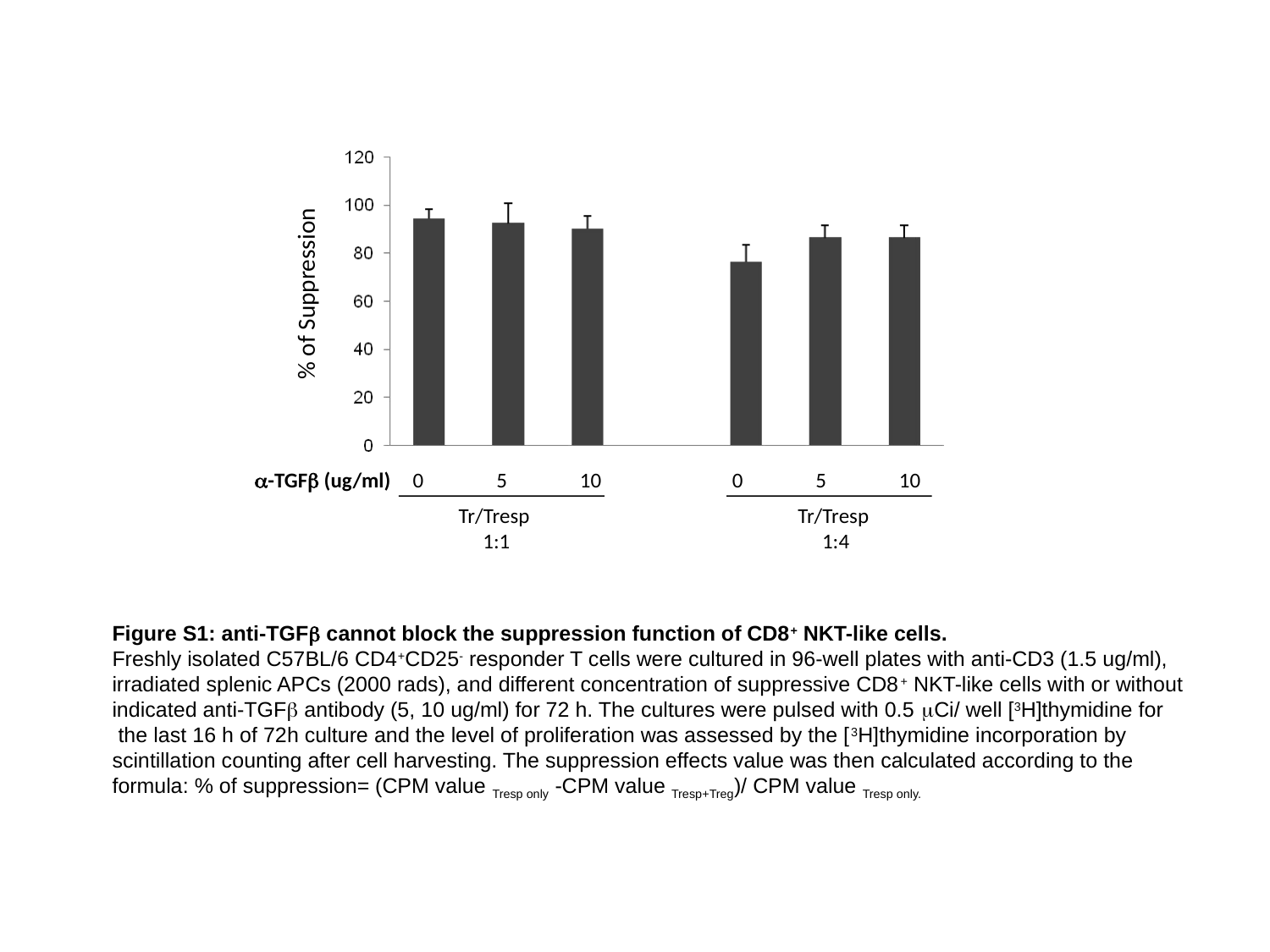

% of Suppression
α-TGFβ (ug/ml)
0 5 10
0 5 10
Tr/Tresp
 1:1
Tr/Tresp
 1:4
Figure S1: anti-TGFβ cannot block the suppression function of CD8+ NKT-like cells.
Freshly isolated C57BL/6 CD4+CD25- responder T cells were cultured in 96-well plates with anti-CD3 (1.5 ug/ml),
irradiated splenic APCs (2000 rads), and different concentration of suppressive CD8+ NKT-like cells with or without
indicated anti-TGFβ antibody (5, 10 ug/ml) for 72 h. The cultures were pulsed with 0.5 Ci/ well [3H]thymidine for
 the last 16 h of 72h culture and the level of proliferation was assessed by the [3H]thymidine incorporation by
scintillation counting after cell harvesting. The suppression effects value was then calculated according to the
formula: % of suppression= (CPM value Tresp only -CPM value Tresp+Treg)/ CPM value Tresp only.

## Slide 2
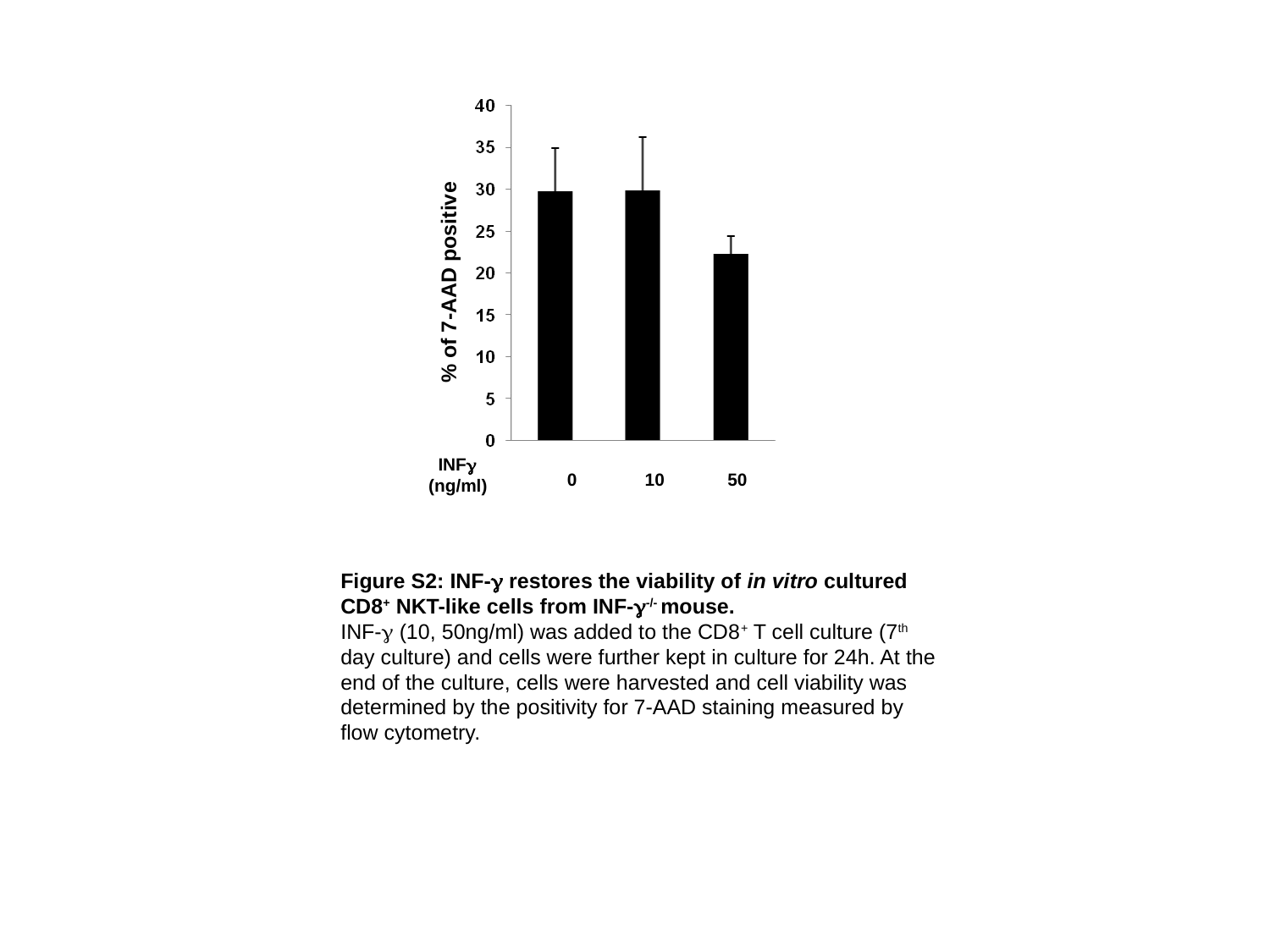

% of 7-AAD positive
 INFγ
(ng/ml)
0 10 50
Figure S2: INF-γ restores the viability of in vitro cultured CD8+ NKT-like cells from INF-γ-/- mouse.
INF-γ (10, 50ng/ml) was added to the CD8+ T cell culture (7th day culture) and cells were further kept in culture for 24h. At the end of the culture, cells were harvested and cell viability was determined by the positivity for 7-AAD staining measured by flow cytometry.
